# Aryloxypropanolamine mitigates Alzheimer‐like phenotype by directly removing amyloid aggregates

**DOI:** 10.1002/alz.088498

**Published:** 2025-01-09

**Authors:** Soljee Yoon, Hee Yang Lee, Illhwan Cho, Donghee Lee, Jisu Shin, Kyeonghwan Kim, Jimin Kim, Hye Yun Kim, Ikyon Kim, YoungSoo Kim

**Affiliations:** ^1^ Yonsei University, Incheon, Incheon Korea, Republic of (South); ^2^ Yonsei Institute of Pharmaceutical Sciences, Incheon Korea, Republic of (South); ^3^ Yonsei University, Incheon Korea, Republic of (South); ^4^ Yonsei Institute of Pharmaceutical Sciences, Incheon, Incheon Korea, Republic of (South)

## Abstract

**Background:**

The accumulation of amyloidogenic proteins is recognized as a primary biomarker, initiator of pathology, and a potential therapeutic target for Alzheimer’s disease (AD). An unbiased screening of a small molecule library was conducted to identify new chemical compounds exhibiting amyloid‐dissociative properties.

**Method:**

The ability of aryloxypropanolamine derivatives to dissociate amyloid‐β (Aβ) aggregates was evaluated through in vitro assays. Subsequent assessments involved immunostaining, immunoblotting, and Morris water maze to examine the anti‐Alzheimer properties of the molecules using 5XFAD and transgenic APP/PS1 mice. Characterization of the target‐ligand interaction was also carried out through peptide mapping assay and constrained docking simulations.

**Result:**

Among 11 derivatives, YIAD002 demonstrated the most significant dissociative effect against β‐sheet‐rich Aβ aggregates. Oral administration of the compound led to a significant reduction in amyloid burden, improved cognitive performance in the Morris water maze, and mitigated key clinical symptoms of AD, including tauopathy, neuroinflammation, and synaptic protein loss. Mechanistic studies indicated that YIAD002 directly interacted with the KLVFFA and IGLMVG domains of Aβ.

**Conclusion:**

Collectively, our results demonstrate the potential of chemical‐driven dissociation of amyloidogenic proteins as a promising disease‐modifying treatment for AD.

